# Minimally Invasive Rehabilitation of a Worn Dentition in a Young Patient: A Case Report

**DOI:** 10.1155/crid/8897041

**Published:** 2026-09-03

**Authors:** Leonardo Mancini, Margherita G. Liguori, Tobias Fischli, Alexis Ioannidis

**Affiliations:** ^1^ Clinic of Reconstructive Dentistry, Center of Dental Medicine, University of Zurich, Zurich, Switzerland, uzh.ch; ^2^ Center for Clinical Research and Evidence Synthesis in Oral Tissue Regeneration (CRITERION), Ann Arbor, Michigan, USA; ^3^ Department of Life, Health and Environmental Science, University of L′aquila, L′Aquila, Italy, univaq.it; ^4^ Dental Technician, Zürich, Switzerland

**Keywords:** adhesive restorations, full-mouth rehabilitation, indirect restoration, lithium disilicate, minimally invasive rehabilitation, partial coverage restorations, tooth wear, vertical dimension of occlusion

## Abstract

Erosive tooth wear (ETW) is increasingly prevalent, particularly among younger populations, and is often associated with dietary habits, including frequent soft‐drink consumption. Patients with advanced tooth wear may report hypersensitivity, impaired mastication, and dissatisfaction with their smile, all of which can negatively affect daily function and quality of life. This case report describes the full‐mouth rehabilitation of a young patient with severe ETW using a minimally invasive approach. Treatment combined digital planning, diagnostic digital wax‐ups, and 3D‐printed preparation guides to support precision and tissue preservation. Lithium disilicate was selected because of its favorable mechanical and optical properties. Its reduced thickness allowed conservative tooth preparation, and the restorations were adhesively cemented according to a carefully planned protocol. Re‐establishing an appropriate vertical dimension of occlusion (VDO) addressed the patient′s functional and esthetic concerns while preserving healthy tooth structure. At the 1‐year follow‐up, the restorations remained stable, supporting the effectiveness of a minimally invasive protocol based on contemporary adhesive techniques and durable ceramic materials.

## 1. Introduction

Erosive tooth wear (ETW) is the irreversible loss of dental hard tissue caused by interacting mechanical and chemical factors [1]. In addition to functional and esthetic impairment, ETW can affect psychological health and social well‐being, especially in young patients [1, 2]. Conventional management of worn dentitions often relied on extensive tooth preparation and full‐coverage indirect restorations [3]. Contemporary minimally invasive dentistry offers alternatives that prioritize preservation of tooth structure while meeting functional and esthetic requirements [4]. From a broader perspective, this conservative approach may also support general oral health.

Although gradual occlusal wear is a physiological process over a lifetime, excessive wear can lead to pulpal pathology, occlusal imbalance, functional impairment, and esthetic deficiencies. Tooth wear may be classified as attrition, abrasion, or erosion according to its main cause [5]. A definitive differential diagnosis may be difficult because these processes frequently coexist [6]. Therefore, identifying contributing factors and evaluating changes in the vertical dimension of occlusion (VDO) are essential. Adaptive responses, such as compensatory tooth eruption and alveolar bone growth, may preserve VDO despite tooth structure loss [7, 8]. When restorative space is limited, as is common in patients with tooth wear, a diagnostic wax‐up can guide treatment planning [9]. Historically, patient tolerance to VDO changes was tested with a diagnostic splint or provisional prosthesis. More recent reviews indicate that this evaluation phase is not always mandatory, and removable occlusal splints may be ineffective for esthetic assessment and may reduce patient satisfaction [1, 10]. To reduce invasiveness, a mock–up‐based guide or splint can enable selective preparation and preserve as much dental tissue as possible [11]. Advances in 3D technology now allow preparation guides based on wax‐ups and mock‐ups to be designed in chairside CAD software and printed.

## 2. Patient Case

**Figure 1:**
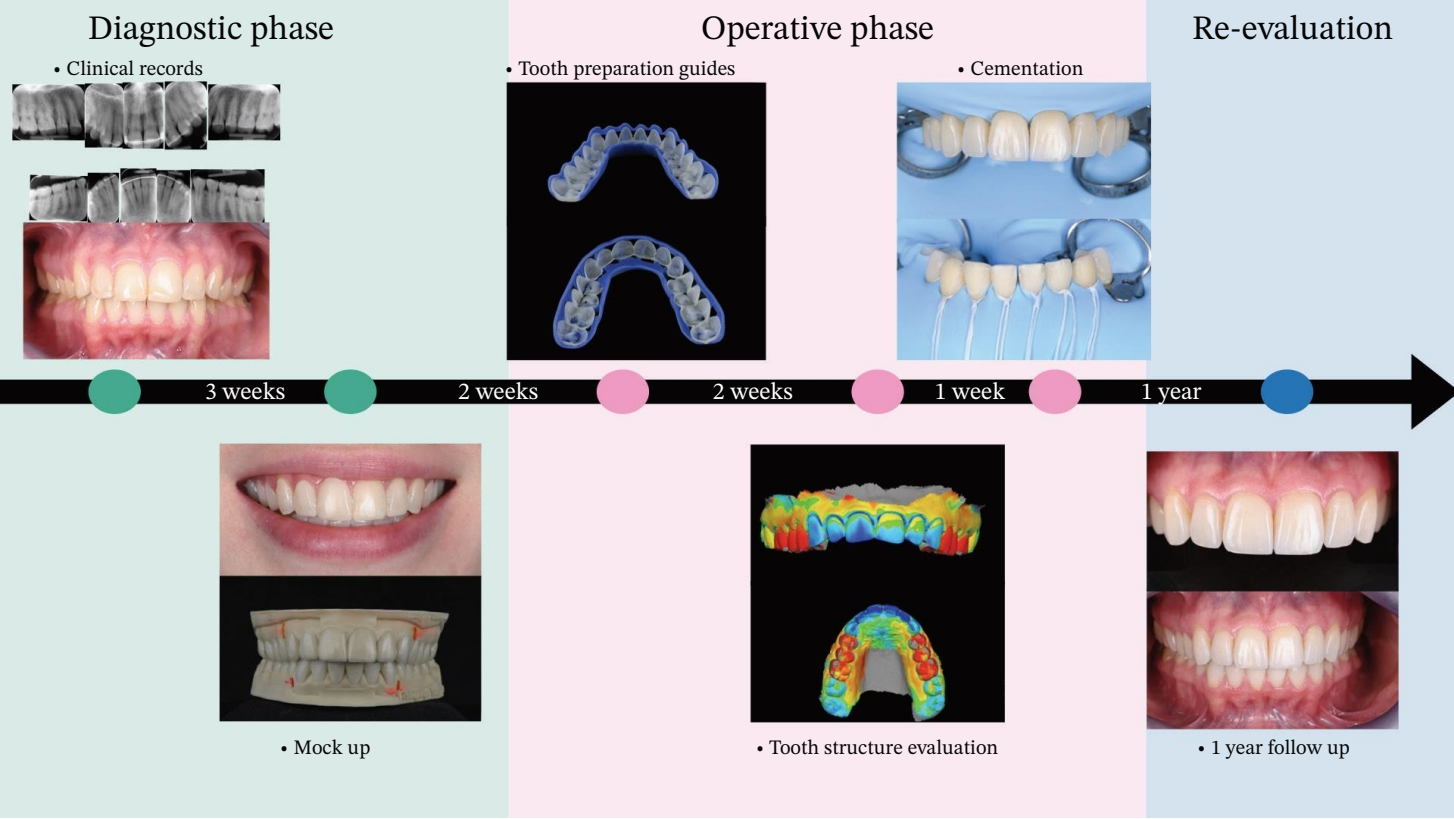
Timeline illustrating the diagnostic workflow and operative treatment sequence.

This case report was prepared in accordance with the CARE (CAse REport) reporting guideline [12]. Ethical approval was not required because the manuscript describes a single anonymized clinical case. The study was conducted in accordance with the Declaration of Helsinki. Written informed consent was obtained from the patient for publication of the clinical information and accompanying images. Figure 1 illustrates the clinical workflow and provides a chronological overview of the diagnostic, preparatory, and restorative procedures performed throughout treatment.

### 2.1. Patient History

A 27‐year‐old female patient was referred to the Clinic for Reconstructive Dentistry at the University of Zurich for comprehensive rehabilitation of severely worn dentition. She reported discomfort during brushing and eating, together with an uncomfortable mandibular resting position. At the initial evaluation, a questionnaire was administered to assess possible bulimia, bruxism and gastroesophageal reflux disease; neither condition was detected. During nutritional anamnesis, the patient reported drinking water with lemon daily for the previous 17 years. The OHIP‐14 questionnaire was also administered to evaluate the effect of her dental condition on quality of life. [13]. Clinical examination revealed marked loss of tooth substance on the buccal and occlusal aspects of the entire dentition. The Basic Erosive Wear Examination (BEWE) yielded a cumulative sextant score of 16, indicating a high risk of erosion [14]. Table 1 showed the percentage and distribution of the scores. The patient had previously undergone a minimally invasive rehabilitation at the age of 16, involving partial‐coverage lithium disilicate restorations of Teeth 12, 11, 21, and 22. Such an early restorative intervention indicates a premature onset of ETW. Following a thorough anamnesis, potential intrinsic and parafunctional etiological factors, including gastroesophageal reflux disease, eating disorders (e.g., bulimia nervosa), and bruxism, were excluded. Consequently, the erosive lesions were considered predominantly attributable to the past 17 years of extrinsic acid exposure particularly water with lemon, which likely played a decisive role in the initiation and progression of the erosive process. Sustained exposure to dietary acids is well recognized as a major risk factor for enamel dissolution and irreversible loss of dental hard tissues, especially in young individuals.

**Table 1:** Basic Erosive Wear Examination (BEWE) index scores at baseline, showing the distribution of erosive tooth wear across the six sextants. The cumulative BEWE score of 16 classified the patient as having a high‐risk erosive wear profile.

Sextant (n. teeth involved)		1 sextant	2 sextant	3 sextant	All upper sextant
BEWE score	0	0	0	0	
	1	0	33.3% [2]	0	11% [2]
	2	33.3% [2]	66.7% [4]	33.3% [2]	44% [8]
	3	66.7% [4]	0	66.7% [4]	44% [8]
BEWE score	0	0	0	0	
	1	0	33.3% [2]	0	1.1% [2]
	2	16.7% [1]	66.7% [4]	66.7% [4]	50% [9]
	3	83.3% [5]	0	33.3% [2]	38.9% [7]
Sextant		6 sextant	5 sextant	4 sextant	All lower sextant

During the following two‐week period, the patient completed a dietary diary to monitor potential erosive risk factors. Review of the diary did not reveal excessive consumption of acidic foods or beverages that could account for the substantial tooth wear observed at the time of assessment. The absence of significant ongoing dietary acid exposure indicated that the erosive process was likely no longer active, thereby allowing progression to definitive restorative treatment with greater confidence in the long‐term stability of the outcome.

### 2.2. Preventive Care

Prior to definitive restorative treatment, a comprehensive preventive protocol was implemented. The patient received individualized oral hygiene and dietary counseling, including instructions to reduce the frequency of acidic food and beverage consumption, avoid prolonged contact with acidic drinks, and postpone toothbrushing for 30–60 min after an erosive challenge. Adequate hydration was also encouraged.

A high‐fluoride toothpaste containing 5000 ppm fluoride (Duraphat 5000 Toothpaste, 1.1% sodium fluoride; Colgate‐Palmolive AG, Therwil, Switzerland) was prescribed for daily use, together with a neutral sodium fluoride mouth rinse. Adherence was assessed at each recall appointment through clinical examination and review of oral‐hygiene practices, dietary habits, and compliance with the prescribed fluoride regimen.

### 2.3. Diagnostic Phase

**Figure 2:**
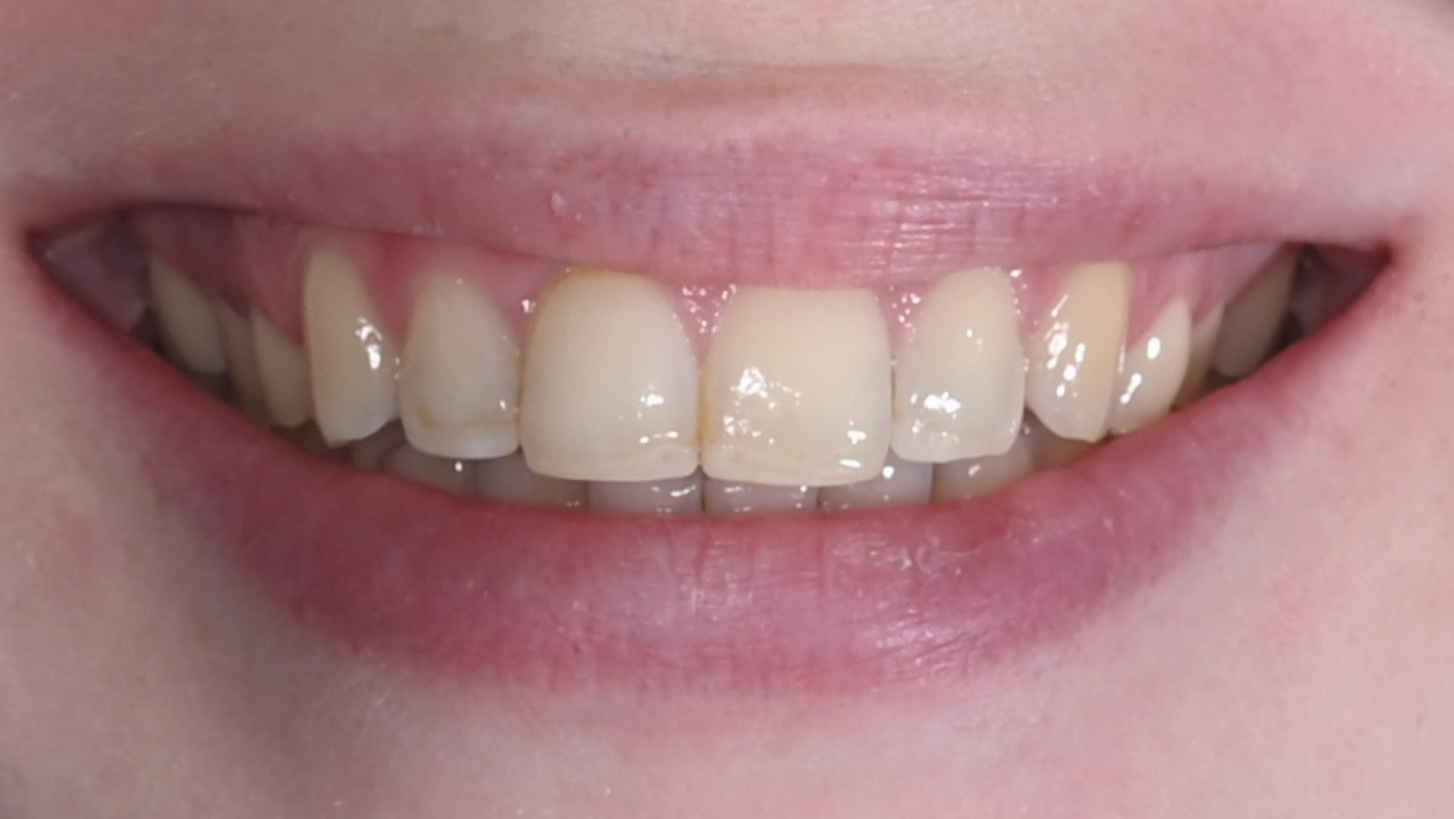
Preoperative smile view showing the existing restorations and incisal wear affecting Teeth 12, 11, 21, and 22.

**Figure 3:**
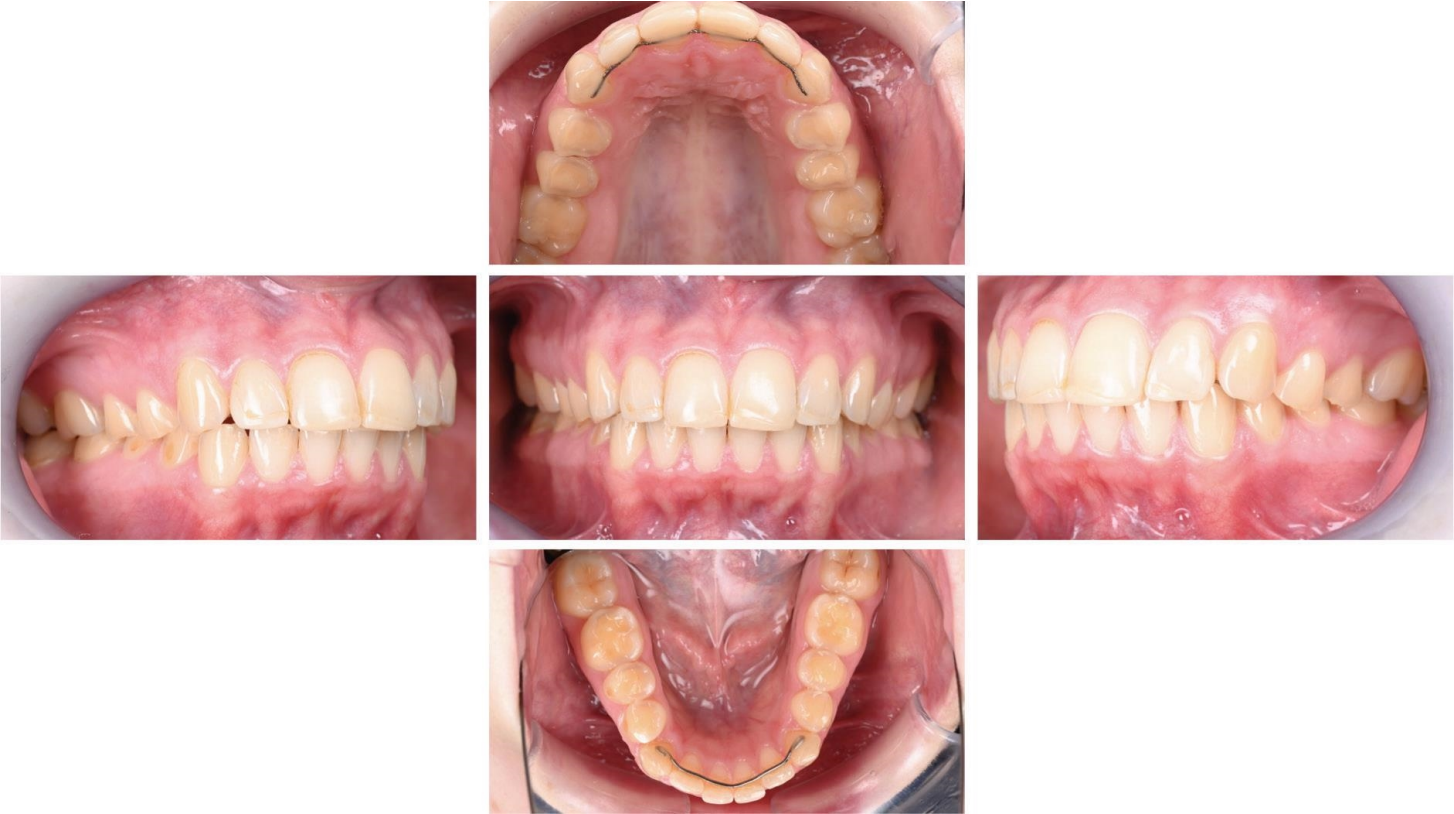
Baseline intraoral photographs including frontal, lateral, and occlusal views. The posterior segments of both the maxillary and mandibular arches exhibit extensive erosive tooth wear.

**Figure 4:**
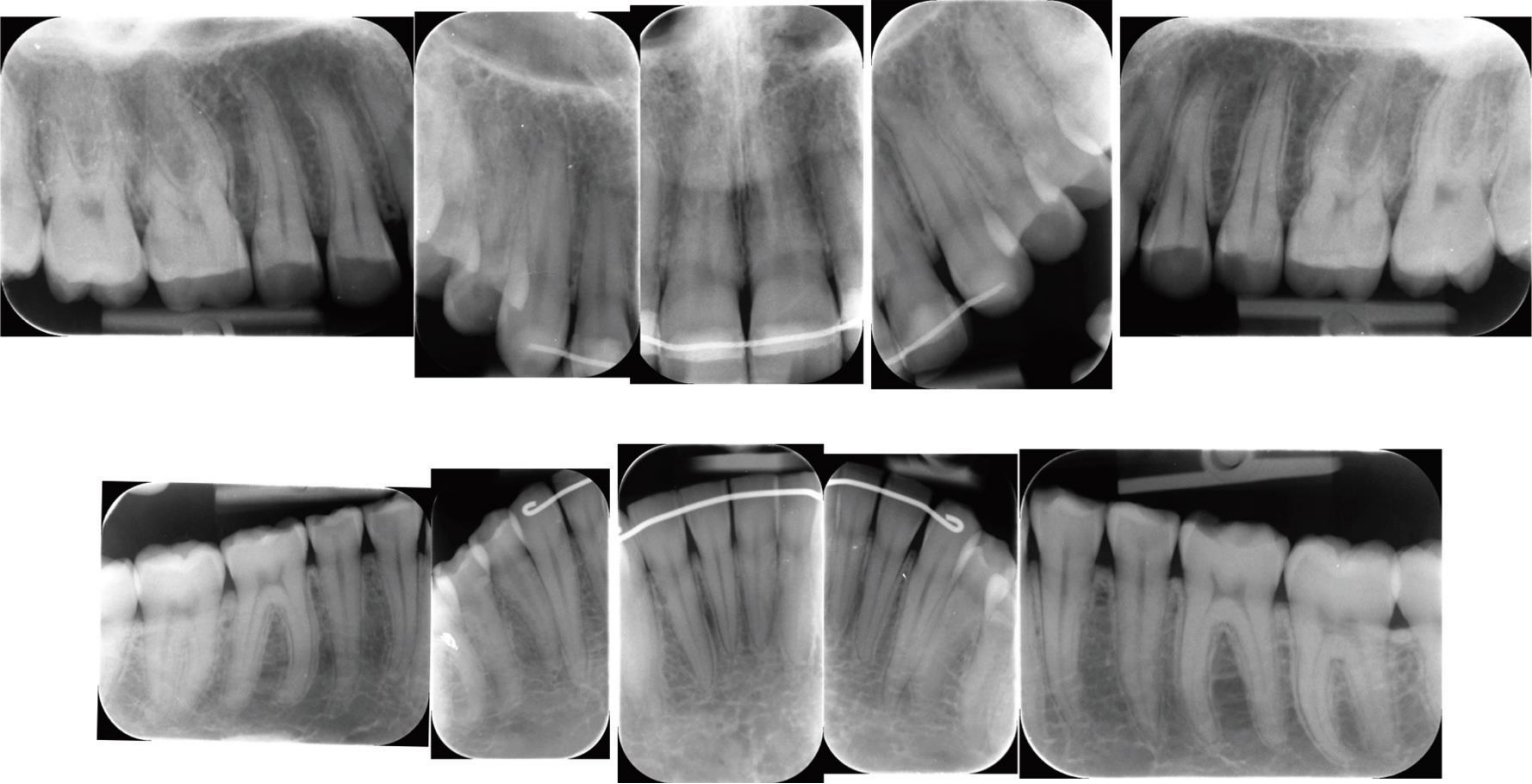
Baseline radiographic assessment.

**Figure 5:**
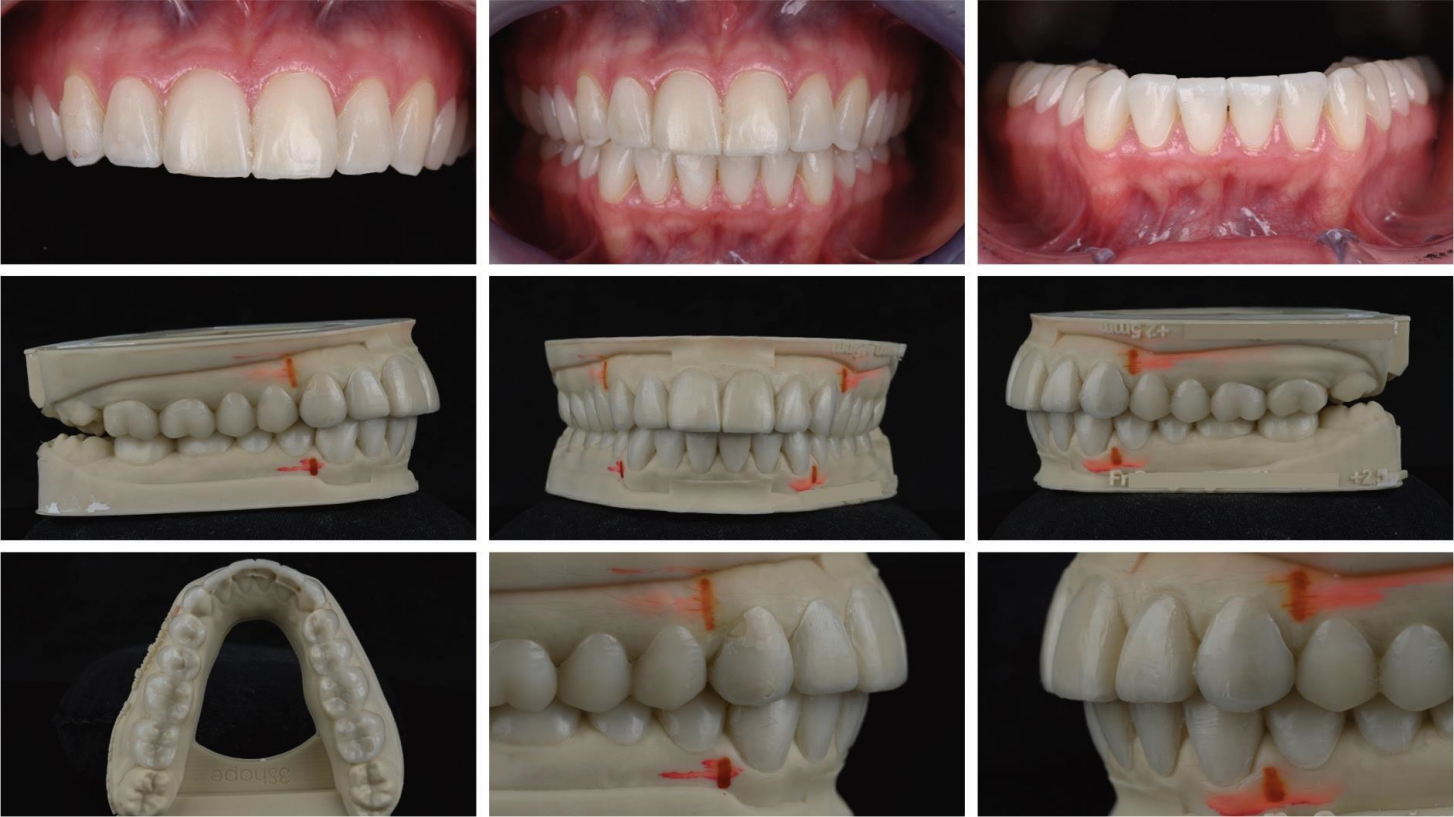
Indirect mock‐up fabricated from milled polymethyl methacrylate (PMMA) for both the maxillary and mandibular arches.

**Figure 6:**
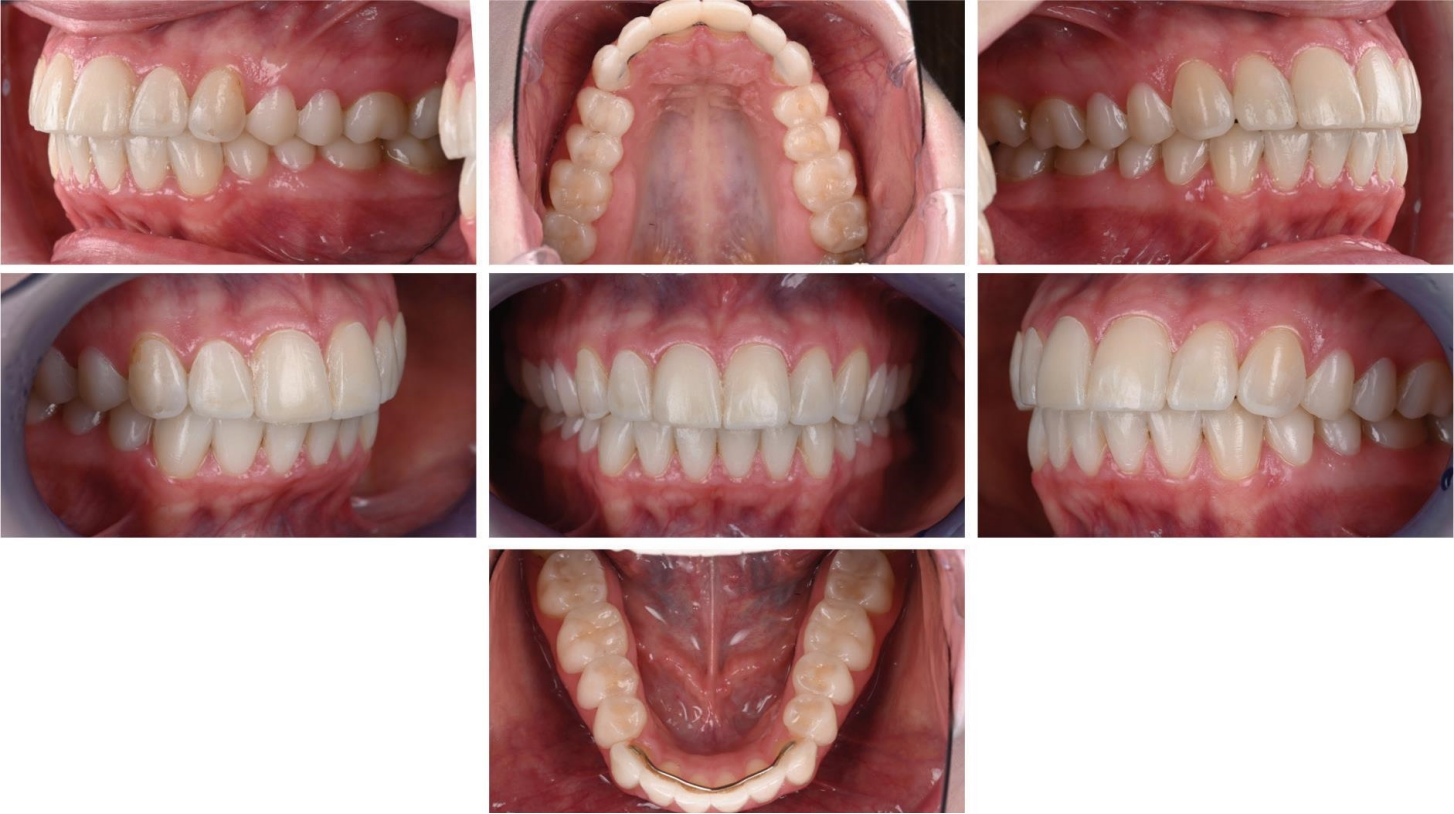
Intraoral overview of the mock‐up demonstrating accurate adaptation, esthetic integration, and an improved smile line.

**Figure 7:**
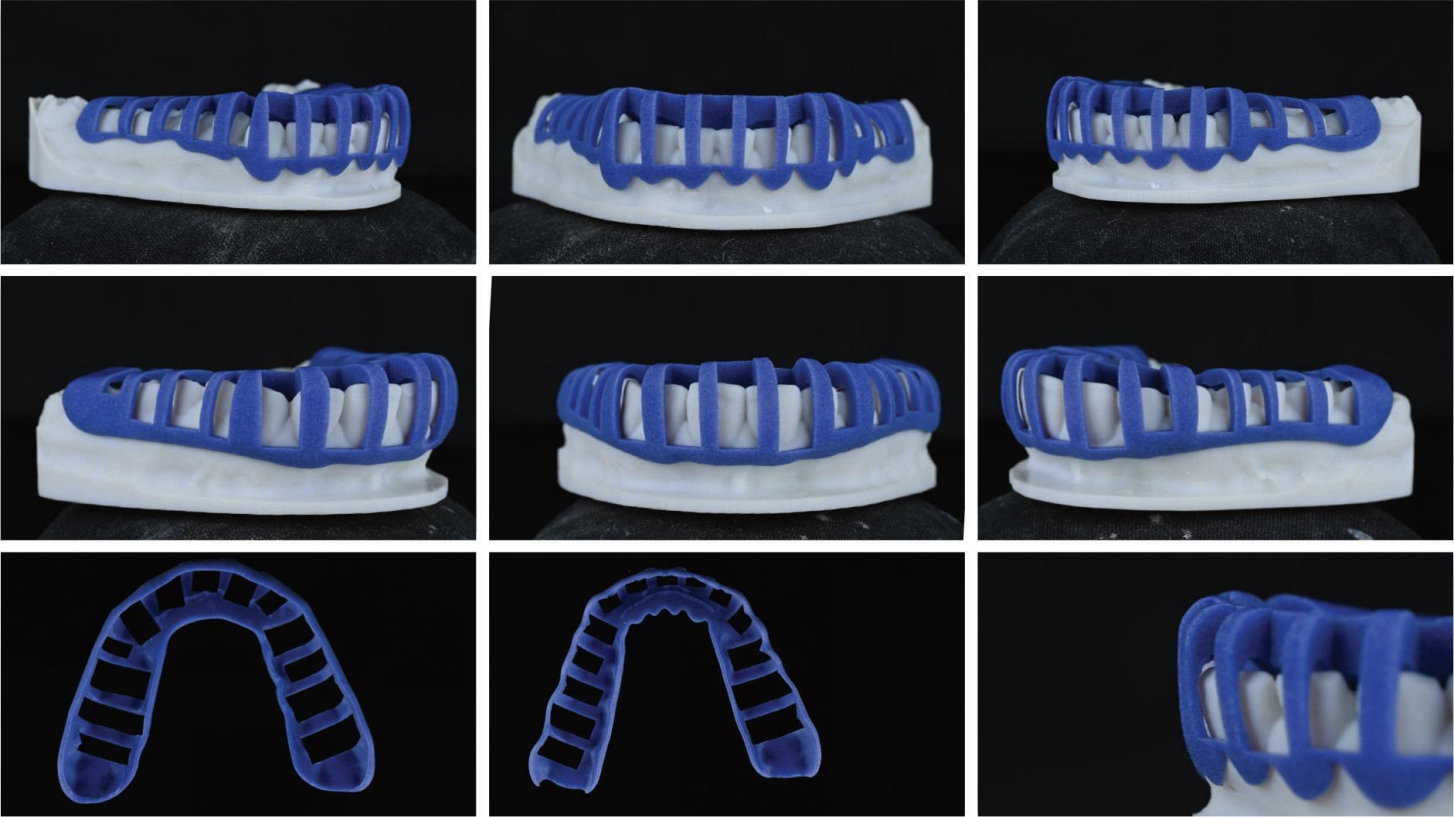
Preparation guides for the maxillary and mandibular arches generated on the initial cast, highlighting areas where no tooth structure reduction was required.

After the initial consultation, the patient′s dental condition was documented with intraoral and extraoral photographs, digital impressions (Trios 3, 3Shape, Copenhagen, Denmark), intraoral radiographs and periodontal parameters such as probing depth (PD), bleeding on probing (BoP), and plaque index (PI) (Figures 2, 3, and 4). In collaboration with a dental technician, a customized digital wax‐up was developed for Teeth 16–26 and 36–46. The wax‐up reproduced correct posterior anatomy, efficient intercuspation, and favorable exposure of the anterior teeth, providing adequate upper lip support. Based on the digital wax‐up and an average‐value digital articulator, the DVO was increased by 2.5 mm between the two dental arches. After photographic and diagnostic evaluation of the indirect milled mock‐up (Figures 5 and 6), the patient was asked to wear it for 7 days, removing it only for eating and drinking, to document potential discomfort, phonetic difficulties, and particularly esthetic concerns. A digital analysis was then performed to determine the required restorative volume and the amount of tooth structure to be removed. Using commercially available CAD software (Medit Link, Medit, Seoul, Yeongdeungpo District, South Korea), a customized digital preparation guide was designed to support minimal preparation of the buccal and occlusal surfaces. The guide had a single‐piece design with two rigid occlusal stops at the second molars and palatal support for stable, reproducible positioning. Buccal windows separated by thin walls allowed direct visualization of the reduction thickness for each tooth on the occlusal, palatal, and buccal aspects (Figure 7).

### 2.4. Operative Phase

**Figure 8:**
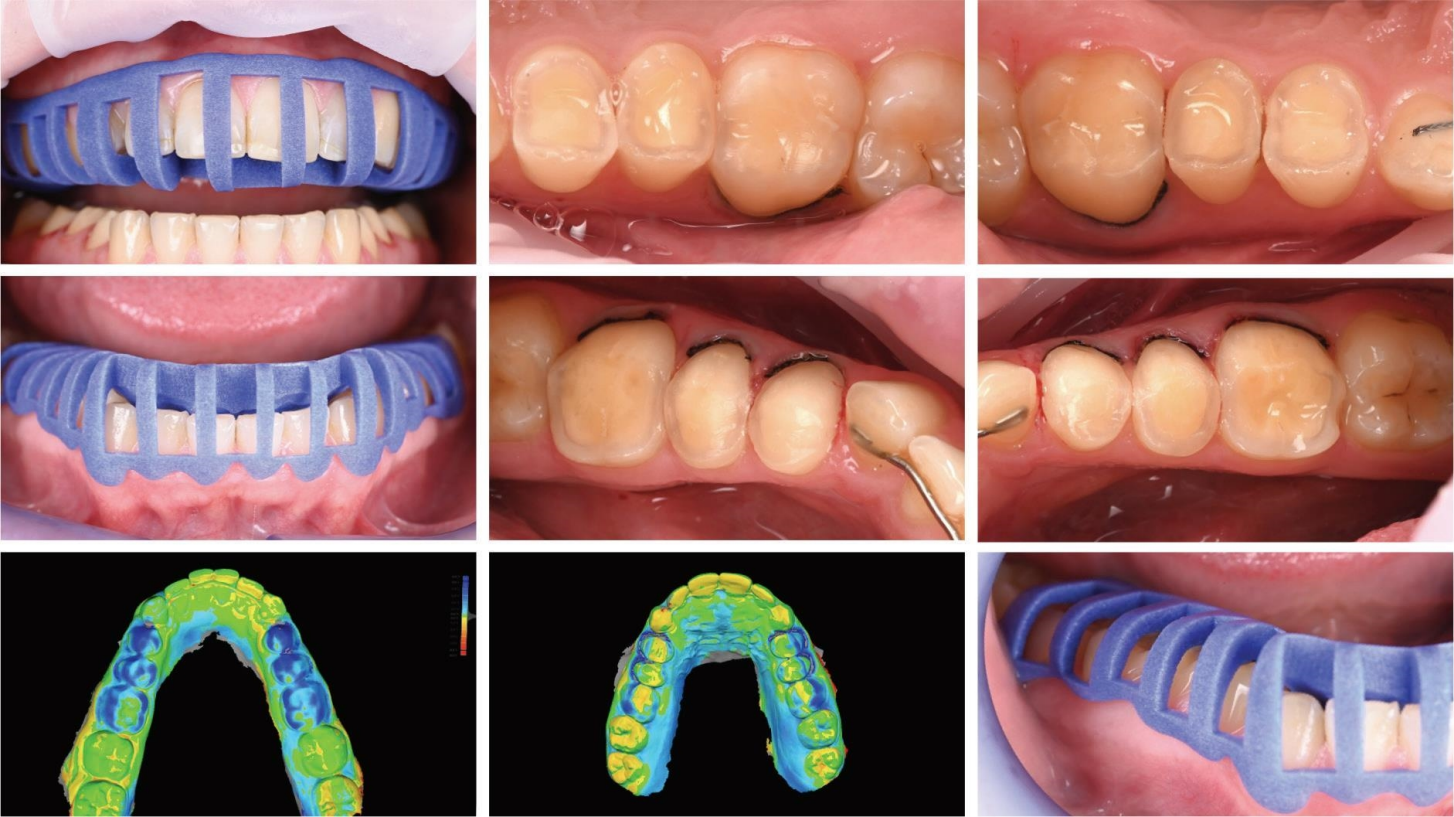
Frontal and lateral intraoral views with the preparation guides in place. Three‐dimensional superimposition analysis showing the amount of tooth structure removed (blue = reduction; green = unchanged surface).

**Figure 9:**
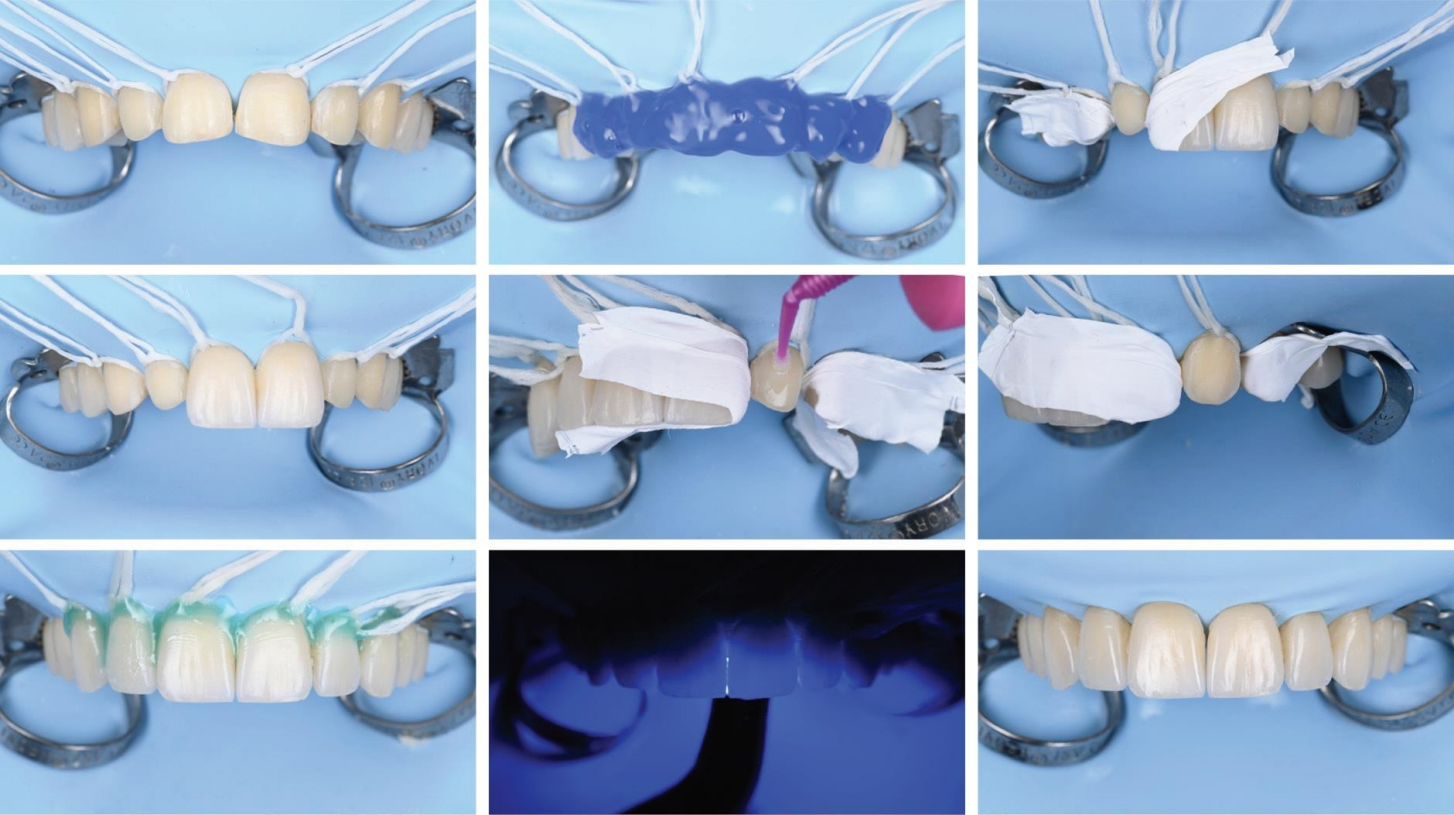
Isolation of the operative field using rubber dam placement in both arches, secured with ligatures.

**Figure 10:**
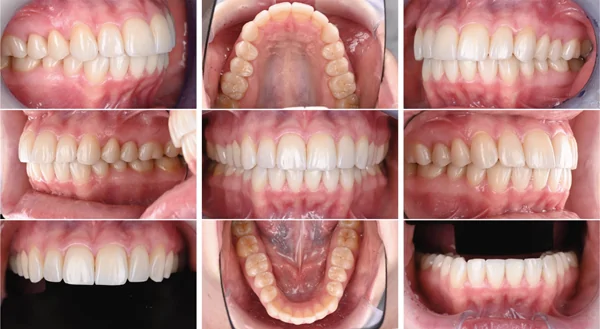
Completed clinical case. Frontal, lateral, and occlusal views of the definitive restorations.

**Figure 11:**
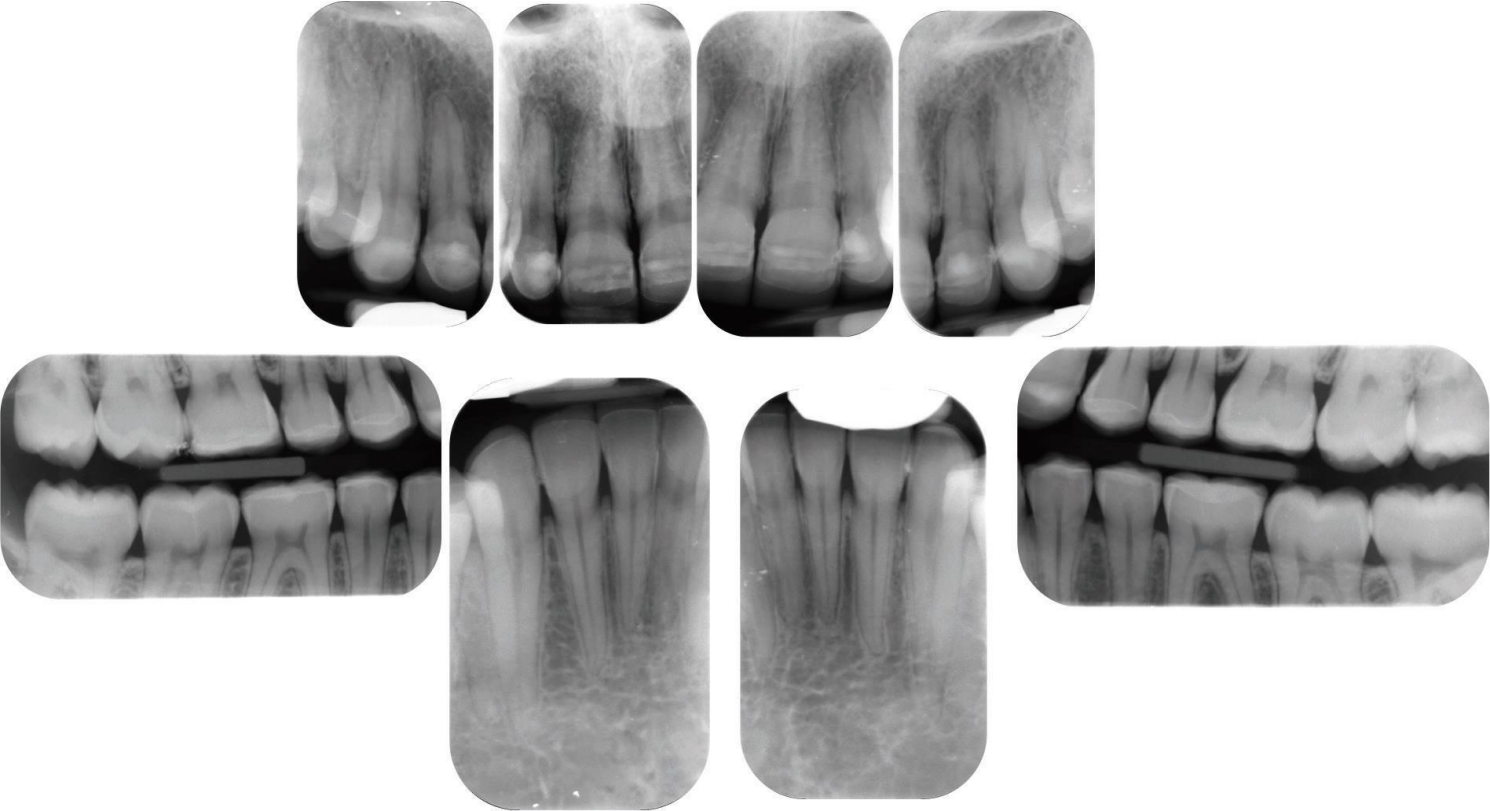
Final radiographic evaluation. Residual cement excesses were identified and subsequently removed following radiographic assessment.

Based on the diagnostic mock‐up, a small gingivectomy was performed around Teeth 14, 15, 24, and 25 to improve the perceived volume and exposure of the future restorations. After this minor modification, the upper and lower posterior sextants were conservatively prepared using digitally fabricated templates (Figure 8). The pre‐existing restorations on the palatal surfaces of Teeth 12, 11, 21, and 22 were removed. Digital scans (Trios 3, 3Shape, Copenhagen, Denmark) were obtained before and after tooth preparation. Superimposition analysis in CAD software (Medit Link, Medit, Seoul, Yeongdeungpo District, South Korea) showed negligible reduction in selected areas of each prepared site, ranging from 0.3 to 0.8 mm, whereas unprepared regions maintained their original dimensions. Burs with different shapes and a standardized 50 *μ*m grain size supported controlled, minimally invasive tooth preparation (Intensiv, Montagnola, Switzerland). Lithium disilicate (IPS e.max Press, Ivoclar Vivadent, Schaan, Liechtenstein) was selected for the full‐mouth restorations. Cementation was preceded by a try‐in appointment to verify restoration fit and accuracy. Before adhesive cementation, the intaglio surfaces of the lithium disilicate restorations were treated according to the Ivoclar protocol. After try‐in, the restorations were cleaned, rinsed, and dried. The internal surfaces were etched with 5% hydrofluoric acid gel (IPS Ceramic Etching Gel, Ivoclar) for 20 s, thoroughly rinsed with water, and air‐dried. A silane‐coupling agent (Monobond Plus, Ivoclar) was then applied to the etched ceramic surfaces, left to react for 60 s, and dispersed with a strong stream of air. A three‐step adhesive protocol etchant, primer, and bonding resin (Ultra‐Etch 35%, Ultradent Products, South Jordan, Utah, United States; Syntac Primer, Syntac Adhesive, and Heliobond, Ivoclar Vivadent) was used to establish an appropriate bonding substrate. The light‐curing resin cement (Variolink Esthetic LC, Ivoclar Vivadent) was selected using two try‐in pastes with different chroma values (Variolink Esthetic Try‐In Paste Neutral and Warm, Ivoclar Vivadent) to match the planned esthetic result. All bonding procedures were performed under complete rubber‐dam isolation (Nic Tone Latex Rubber Dam 6 × 6, Medium Gauge Blue 36/Bx, MDC Dental/Reko Dental, Gardena, California, United States) to avoid contamination during conditioning and cementation (Figure 9). Once the restorations were correctly seated, excess cement was initially removed with microbrushes. A brief light‐polymerization step increased cement consistency, allowing easier removal with a scaler. After final polymerization, the restorations were polished with dark‐blue and light‐blue rubber polishing instruments (OptraGloss Polisher, Ivoclar Vivadent) (Figure 10). Radiographs were taken to detect and, if necessary, immediately remove any residual cement (Figure 11).

Following the 2.5‐mm increase in VDO, the definitive restorations were designed to establish a mutually protected occlusal scheme. In maximum intercuspation, simultaneous and evenly distributed bilateral posterior contacts were achieved, with light and stable contacts on the anterior teeth. During lateral mandibular excursions, canine guidance was established to ensure immediate disclusion of the posterior teeth, thereby minimizing lateral loading on the posterior restorations. In protrusive movements, anterior guidance was provided by the maxillary and mandibular incisors, resulting in complete posterior disclusion.

Occlusal contacts were initially assessed clinically using articulating paper of different thicknesses (40 and 12 *μ*m) to identify both gross and fine contact points. The intensity and distribution of contacts were further evaluated through shimstock foil (8 *μ*m) testing to verify the presence of stable holding contacts in maximum intercuspation and the absence of undesirable contacts during excursive movements. Final occlusal adjustments were performed intraorally until uniform bilateral contact distribution and smooth, interference‐free mandibular excursions were achieved. The occlusal scheme was subsequently reassessed at follow‐up appointments to confirm adaptation to the increased VDO and maintenance of occlusal stability.

## 3. Results

At the 1‐year follow‐up, the OHIP‐14 questionnaire was repeated to assess the influence of the patient′s dental condition on quality of life. The results indicated a substantial improvement in oral–health‐related well‐being, with the score decreasing from 32/56 to 6/56. Periodontal parameters were stable over time with no increase in PD, BoP, and plaque index (PI). All lithium disilicate tabletop restorations and veneers remained clinically and esthetically satisfactory. According to the modified USPHS/FDI evaluation criteria, marginal adaptation was rated as alpha/excellent for all restorations, as the margins remained continuous with the tooth structure, without detectable gaps, overhangs, marginal staining, or signs of restoration debonding. Furthermore, no evidence of secondary caries, fracture, chipping, or loss of retention was observed. The integrity of the restoration‐tooth interface was maintained throughout the observation period, indicating favorable clinical performance and stability of the adhesive bonding protocol.

Regarding color match and translucency, all restorations also received an alpha/excellent rating. The lithium disilicate restorations maintained their original shade and optical properties, exhibiting excellent integration with the adjacent dentition. No clinically perceptible discoloration, staining, mismatch in hue, chroma, or value, or loss of translucency was detected. The restorations continued to provide a natural and harmonious appearance, contributing to the overall esthetic success of the full‐mouth rehabilitation.

Overall, the clinical and esthetic outcomes remained stable after 1 year of function, with no restoration requiring polishing, repair, adjustment, or replacement. Patient satisfaction with both function and appearance was high, supporting the predictability and durability of lithium disilicate veneers and tabletop restorations in comprehensive adhesive full‐mouth rehabilitation.

Table 2 summarized the baseline and 1‐year follow‐up clinical data.

**Table 2:** Clinical parameters recorded at baseline and follow‐up. The results demonstrate an improvement in Oral Health Impact Profile (OHIP‐14) scores, accompanied by stable periodontal parameters throughout the observation period.

	PD	BOP	PI	OHIP‐14
Baseline	2.3 ± 0.72	8.3%	35.4%	32/56
1 year follow‐up	2.4 ± 0.70	7.2%	18.3%	6/56

## 4. Discussion

Patients with tooth wear often present permanent anatomical alterations that may cause long‐term functional and esthetic challenges. To obtain durable outcomes, management should extend beyond short‐term symptomatic measures. [5, 8, 15, 16]. Although diagnostic criteria for ETW vary widely, recent reports suggest that erosion‐related tooth wear is increasingly prevalent among children and young adults. [17–19]. In younger individuals, soft drink consumption has been identified as an important factor associated with dental erosion and tooth wear [16]. In the present case, the patient was 27 years old, and the only objective etiological factor identified was long‐term daily consumption of water with lemon. Nevertheless, severe pathological tooth wear is often multifactorial. The patient reported discomfort during brushing, hypersensitivity, and dissatisfaction with the appearance of the anterior teeth. Reduced enamel thickness exposes dentin, increasing permeability to thermal and chemical stimuli. Tissue loss also results in shorter, darker anterior teeth, and a less pleasing smile. The patient reported no temporomandibular joint disorder, masticatory dysfunction, or bruxism. Treatment therefore aimed to restore posterior anatomy and function while creating a natural, attractive anterior appearance, an important objective in a young patient.

Several treatment strategies have been proposed for excessively worn dentitions. Clinicians should prioritize preservation of the already reduced tooth structure, maintenance of tooth vitality, and creation of adequate interocclusal space for the restorative material [4, 6, 7, 20–22]. Advances in reinforced ceramic materials and adhesive techniques have made minimally invasive partial‐coverage restorations with increased VDO a viable alternative to full‐coverage crowns in extensively worn dentitions [8, 22–25]. In this case, an indirect PMMA shell‐type mock‐up was used before definitive treatment. Although recent systematic reviews suggest that this evaluation phase can be omitted in selected situations, it remains useful for assessing patient comfort, function, esthetics, and the proposed occlusal scheme [10, 23]. The stepwise approach may also help less experienced clinicians and laboratory teams refine the treatment plan before definitive restorations are fabricated.

Lithium disilicate was selected because it is a reliable material for both full crowns and partial restorations. More than many other restorative materials, lithium disilicate benefits from conservative preparation that preserves enamel as the preferred bonding substrate and optimizes adhesion [4, 8, 11, 24–28]. Excessive preparation with dentin exposure can increase the risk of microleakage and adhesive fractures [29–32]. When bonded to enamel, monolithic etchable ceramic restorations can be fabricated with reduced thickness while providing results comparable with feldspathic ceramic restorations, which typically require 1.5–2.0 mm of thickness [33]. Direct or indirect composite resin restorations may also represent a conservative treatment alternative, particularly in younger patients, cases with limited structural loss, financial constraints, or when a more reversible approach is desired. However, compared with lithium disilicate, composite materials generally exhibit lower wear resistance, color stability, and long‐term mechanical performance, although they offer the advantages of easier repair and reduced biological cost [34, 35].

The mock‐up was used to preview the planned occlusal and esthetic changes, particularly because the VDO was modified [10, 36]. It allowed both clinician and patient to evaluate the proposed treatment before fabrication of the definitive restorations. The optimal duration for mock‐up wear remains debated, with recommendations ranging from a few days to several weeks depending on the clinical protocol and patient adaptation [27, 37].

This case highlights the complementary roles of the mock‐up and the preparation guide. The mock‐up supported evaluation of the intended treatment outcome, whereas the guide controlled the amount of tooth reduction required. The shape and volume of the final restorations were determined from the digital wax‐up. The preparation guide used in this case was digitally designed and fabricated using CAM technology, following methods previously described in the literature [4, 9, 22, 36]. Although the technique considered the final veneer contours, it cannot be classified as fully mock–up‐driven preparation. It more closely resembles a silicone‐index‐guided approach, which has been reported to be less conservative than mock–up‐driven preparation [27]. However, unlike a silicone index, this guide was made from a rigid, noncompressible material; the authors therefore consider it potentially more precise and supportive of conservative tooth reduction.

Following full‐mouth rehabilitation of worn dentition, patients may experience meaningful improvements in quality of life, as observed in this case. Restored function, esthetics, and oral health can reduce chewing discomfort and esthetic concerns that affect confidence. Published studies indicate that restoring the VDO and using durable restorative materials can provide long‐term functional and psychological benefits [38, 39]. Reports of comparable cases support these outcomes, showing that well‐planned rehabilitation can lead to sustained satisfaction with appearance and dental function [38, 39].

Control of etiological factors was considered essential to the long‐term prognosis of the adhesive rehabilitation. Dietary modification and maintenance of salivary flow reduce erosive challenges and support acid clearance and buffering [40]. Delaying toothbrushing for 30–60 min was advised to limit abrasion of acid‐softened tissues, although evidence for a specific waiting period remains inconclusive [41, 42]. High‐fluoride toothpaste and a neutral sodium fluoride rinse were prescribed to increase topical fluoride exposure, reduce further tissue loss, and manage dentin hypersensitivity [43, 44]. Continued adherence and recall monitoring were therefore integral to protecting the remaining tooth structure and restorative margins.

The present case report is limited by its single‐patient design and the relatively short 1‐year follow‐up, which precludes conclusions regarding the long‐term clinical performance of the rehabilitation. Nevertheless, its strengths include the comprehensive documentation of a fully digital, minimally invasive workflow combined with preventive management and adhesive rehabilitation, demonstrating stable functional, biological, and esthetic outcomes. Further prospective studies with larger cohorts and extended follow‐up are required to validate these findings.

## 5. Conclusions

This case report supports the cited literature and shows that minimally invasive procedures can be reliable in complete rehabilitation of worn dentition. The approach achieved favorable esthetic and functional outcomes while minimizing additional reduction of dental tissue that had already been compromised by pathological wear.

## Author Contributions

L.M., M.G.L., and A.I. were involved in the conception and design of the study. L.M. and M.G.L. collected the data. L.M. performed the data analysis and interpretation. L.M., M.G.L., A.I., and T.F. contributed to the final drafting of the work.

## Funding

No funding was received for this manuscript. Open access publishing facilitated by Universitat Zurich, as part of the Wiley ‐ Universitat Zurich agreement via the Consortium Of Swiss Academic Libraries.

## Disclosure

All authors have read and approved the final version of the manuscript. L.M. had full access to all study data and takes complete responsibility for data integrity and the accuracy of the analysis.

## Conflicts of Interest

The authors declare no conflicts of interest.

## Supporting information


**Supporting Information** Additional supporting information can be found online in the Supporting Information section. 

## Data Availability

The data that support the findings of this study are available from the corresponding author upon reasonable request.

## References

[1] Patel J. and Baker S. R. , Is Toothwear Associated With Oral Health Related Quality of Life in Adults in the UK?, Community Dental Health. (2020) 37, no. 3, 174–179, 10.1922/CDH_00026Patel06, 32212433. 32212433

[2] Smith B. G. , Bartlett D. W. , and Robb N. D. , The Prevalence, Etiology and Management of Tooth Wear in the United Kingdom, Journal of Prosthetic Dentistry. (1997) 78, no. 4, 367–372, 10.1016/S0022-3913(97)70043-X, 9338867. 9338867

[3] Malkoc M. A. , Sevimay M. , and Yaprak E. , The Use of Zirconium and Feldspathic Porcelain in the Management of the Severely Worn Dentition: A Case Report, European Journal of Dentistry. (2009) 3, no. 1, 75–80, 10.1055/s-0039-1697410, 19262736. 19262736 PMC2647964

[4] Edelhoff D. , Liebermann A. , Beuer F. , Stimmelmayr M. , and Guth J. F. , Minimally Invasive Treatment Options in Fixed Prosthodontics, Quintessence Int. (2016) 47, no. 3, 207–216, 10.3290/j.qi.a35115, 26925471. 26925471

[5] Song M. Y. , Park J. M. , and Park E. J. , Full Mouth Rehabilitation of the Patient With Severely Worn Dentition: A Case Report, journal of Advanced Prosthodontics. (2010) 2, no. 3, 106–110, 10.4047/jap.2010.2.3.106, 21165279. 21165279 PMC2994694

[6] Loomans B. , Opdam N. , Attin T. , Bartlett D. , Edelhoff D. , Frankenberger R. , Benic G. , Ramseyer S. , Wetselaar P. , Sterenborg B. , Hickel R. , Pallesen U. , Mehta S. , Banerji S. , Lussi A. , and Wilson N. , Severe Tooth Wear: European Consensus Statement on Management Guidelines, Journal of Adhesive Dentistry. (2017) 19, no. 2, 111–119, 10.3290/j.jad.a38102, 28439579. 28439579

[7] Debbarma L. and Sharma V. , Full Mouth Rehabilitation for a Patient With Generalized Attrition: The Hobo Technique in Action, Cureus. (2024) 16, no. 1, e51933, 10.7759/cureus.51933, 38333449. 38333449 PMC10851815

[8] Moslehifard E. , Nikzad S. , Geraminpanah F. , and Mahboub F. , Full-Mouth Rehabilitation of a Patient With Severely Worn Dentition and Uneven Occlusal Plane: A Clinical Report, Journal of Prosthodontics: Implant, Esthetic and Reconstructive Dentistry. (2012) 21, no. 1, 56–64, 10.1111/j.1532-849X.2011.00765.x, 21981748. 21981748

[9] Villalobos-Tinoco J. , Jurado C. A. , Rojas-Rueda S. , and Fischer N. G. , Additive Wax-Up and Diagnostic Mockup As Driving Tools for Minimally Invasive Veneer Preparations, Cureus. (2022) 14, no. 7, e27402, 10.7759/cureus.27402, 36046283. 36046283 PMC9418764

[10] Chantler J. , Strasding M. , Strauss F. , Ioannidis A. , and Naenni N. , Importance of an Evaluation Phase When Increasing the Occlusal Vertical Dimension: A Systematic Review, Journal of Esthetic and Restorative Dentistry. (2025) 37, no. 3, 669–689, 10.1111/jerd.13331, 39404129. 39404129 PMC12076109

[11] Robles M. , Jurado C. A. , Floriani F. , Rojas-Rueda S. , Garcia P. , and Azpiazu-Flores F. X. , 3D-Printed Tooth Reduction Guide for Minimally Invasive Veneer Preparations. A Case Report, International Journal of Esthetic Dentistry. (2024) 19, no. 4, 348–359, 39422269. 39422269

[12] Gagnier J. J. , Kienle G. , Altman D. G. , Moher D. , Sox H. , Riley D. , and the CARE Group , The CARE Guidelines: Consensus-Based Clinical Case Reporting Guideline Development, Global Advances in Health and Medicine. (2013) 2, no. 5, 38–43, 10.7453/gahmj.2013.008, 24155002. PMC3833570 24416692

[13] Slade G. D. , Derivation and Validation of a Short-Form Oral Health Impact Profile, Community Dentistry and oral Epidemiology. (1997) 25, no. 4, 284–290, 10.1111/j.1600-0528.1997.tb00941.x, 9332805. 9332805

[14] Bartlett D. , Ganss C. , and Lussi A. , Basic Erosive Wear Examination (BEWE): A New Scoring System for Scientific and Clinical Needs, Clinical Oral Investigations. (2008) 12, no. S1, 65–68, 10.1007/s00784-007-0181-5. PMC2238785 18228057

[15] Lassmann L. , Pioro A. , Calatrava J. , and Wang H. L. , A Clinical Algorithm for Full-Mouth Rehabilitation in Patients With Generalized Severe Tooth Wear: A Case Report, Clinical and Experimental Dental Research. (2026) 12, no. 2, e70349, 10.1002/cre2.70349, 42035476. 42035476 PMC13110831

[16] Okunseri C. , Okunseri E. , Gonzalez C. , Visotcky A. , and Szabo A. , Erosive Tooth Wear and Consumption of Beverages Among Children in the United States, Caries Research. (2011) 45, no. 2, 130–135, 10.1159/000324109, 21430382. 21430382

[17] Campus G. , Niu J. Y. , Sezer B. , and Yu O. Y. , Prevention and Management of Dental Erosion and Decay, BMC Oral Health. (2024) 24, no. 1, 10.1186/s12903-024-04257-y, 38632545. PMC11025157 38632545

[18] Jaeggi T. and Lussi A. , Prevalence, Incidence and Distribution of Erosion, Monographs in Oral Science. (2014) 25, 55–73, 10.1159/000360973. 24993258

[19] Kanzow P. , Wegehaupt F. J. , Attin T. , and Wiegand A. , Etiology and Pathogenesis of Dental Erosion, Quintessence International. (2016) 47, no. 4, 275–278, 10.3290/j.qi.a35625, 27022647. 27022647

[20] Edelhoff D. , Guth J. F. , Erdelt K. , Brix O. , and Liebermann A. , Clinical Performance of Occlusal Onlays Made of Lithium Disilicate Ceramic in Patients With Severe Tooth Wear Up to 11 Years, Dental Materials. (2019) 35, no. 9, 1319–1330, 10.1016/j.dental.2019.06.001, 31256912. 31256912

[21] Fan J. , Wang B. , Wang L. , Xu B. , Wang L. , Wang C. , and Fu B. , Clinical Performance of Minimally Invasive Full-Mouth Rehabilitation Using Different Materials and Techniques for Patients With Moderate to Severe Tooth Wear: A Systematic Review and Meta-Analysis, Clinical Oral Investigations. (2025) 29, no. 2, 10.1007/s00784-025-06181-z, 39875663. 39875663

[22] Fradeani M. , Barducci G. , and Bacherini L. , Esthetic Rehabilitation of a Worn Dentition With a Minimally Invasive Prosthetic Procedure (MIPP), International Journal of Esthetic Dentistry. (2016) 11, no. 1, 16–35, 26835522. 26835522

[23] Goldstein G. , Goodacre C. , and Mac G. K. , Occlusal Vertical Dimension: Best Evidence Consensus Statement, Journal of Prosthodontics. (2021) 30, no. S1, 12–19, 10.1111/jopr.13315, 33783090. 33783090

[24] Ma L. , Guess P. C. , and Zhang Y. , Load-Bearing Properties of Minimal-Invasive Monolithic Lithium Disilicate and Zirconia Occlusal Onlays: Finite Element and Theoretical Analyses, Dental Materials. (2013) 29, no. 7, 742–751, 10.1016/j.dental.2013.04.004, 23683531. 23683531 PMC3698988

[25] Spitznagel F. A. , Prott L. S. , Hoppe J. S. , Manitckaia T. , Blatz M. B. , Zhang Y. , Langner R. , and Gierthmuehlen P. C. , Minimally Invasive CAD/CAM lithium Disilicate Partial-Coverage Restorations Show Superior In-Vitro Fatigue Performance Than Single Crowns, Journal of Esthetic and Restorative Dentistry. (2024) 36, no. 1, 94–106, 10.1111/jerd.13169, 38009505. 38009505 PMC10872741

[26] Gehrt M. , Wolfart S. , Rafai N. , Reich S. , and Edelhoff D. , Clinical Results of Lithium-Disilicate Crowns After Up to 9 Years of Service, Clinical Oral Investigations. (2013) 17, no. 1, 275–284, 10.1007/s00784-012-0700-x, 22392163. 22392163

[27] Gurel G. , Morimoto S. , Calamita M. A. , Coachman C. , and Sesma N. , Clinical Performance of Porcelain Laminate Veneers: Outcomes of the Aesthetic Pre-Evaluative Temporary (APT) Technique, International Journal of Periodontics & Restorative Dentistry. (2012) 32, no. 6, 625–635, 23057051. 23057051

[28] Sadowsky S. J. , An Overview of Treatment Considerations for Esthetic Restorations: A Review of the Literature, Journal of Prosthetic Dentistry. (2006) 96, no. 6, 433–442, 10.1016/j.prosdent.2006.09.018, 17174661. 17174661

[29] Blatz M. B. , Sadan A. , and Kern M. , Resin-Ceramic Bonding: A Review of the Literature, Journal of Prosthetic Dentistry. (2003) 89, no. 3, 268–274, 10.1067/mpr.2003.50, 12644802. 12644802

[30] Blatz M. B. , Vonderheide M. , and Conejo J. , The Effect of Resin Bonding on Long-Term Success of High-Strength Ceramics, Journal of Dental Research. (2018) 97, no. 2, 132–139, 10.1177/0022034517729134, 28876966. 28876966 PMC6429574

[31] Magne P. and Douglas W. H. , Porcelain Veneers: Dentin Bonding Optimization and Biomimetic Recovery of the Crown, International Journal of Prosthodontics. (1999) 12, no. 2, 111–121, 10371912. 10371912

[32] Samartzi T. K. , Papalexopoulos D. , Sarafianou A. , and Kourtis S. , Immediate Dentin Sealing: A Literature Review, Clinical, Cosmetic and Investigational Dentistry. (2021) 13, 233–256, 10.2147/CCIDE.S307939, 34188553. 34188553 PMC8232880

[33] Lassmann L. , Calamita M. A. , and Manfredini D. , Myths Surrounding Vertical Dimension of Occlusion in Restorative Dentistry: A Scoping Review, Journal of Esthetic and Restorative Dentistry. (2025) 37, no. 1, 94–105, 10.1111/jerd.13303, 39189329. 39189329

[34] Gresnigt M. M. M. , Cune M. S. , Jansen K. , van der Made S. A. M. , and Ozcan M. , Randomized Clinical Trial on Indirect Resin Composite and Ceramic Laminate Veneers: Up to 10-Year Findings, Journal of Dentistry. (2019) 86, 102–109, 10.1016/j.jdent.2019.06.001, 31181242. 31181242

[35] Lim T. W. , Tan S. K. , Li K. Y. , and Burrow M. F. , Survival and Complication Rates of Resin Composite Laminate Veneers: A Systematic Review and Meta-Analysis, Journal of Evidence-Based Dental Practice. (2023) 23, no. 4, 101911, 10.1016/j.jebdp.2023.101911, 38035903. 38035903

[36] Lee H. , Fehmer V. , Kwon K. R. , Burkhardt F. , Pae A. , and Sailer I. , Virtual Diagnostics and Guided Tooth Preparation for the Minimally Invasive Rehabilitation of a Patient With Extensive Tooth Wear: A Validation of a Digital Workflow, Journal of Prosthetic Dentistry. (2020) 123, no. 1, 20–26, 10.1016/j.prosdent.2018.11.023, 31079881. 31079881

[37] Farias-Neto A. , de Medeiros F. C. D. , Vilanova L. , Simonetti Chaves M. , Batista F. , and de Araujo J. J. , Tooth Preparation for Ceramic Veneers: When Less is More, International Journal of Esthetic Dentistry. (2019) 14, no. 2, 156–164. 31061996

[38] Ferrando Cascales Á. , Sauro S. , Hirata R. , Astudillo-Rubio D. , Ferrando Cascales R. , Agustín-Panadero R. , and Delgado-Gaete A. , Total Rehabilitation Using Adhesive Dental Restorations in Patients With Severe Tooth Wear: A 5-Year Retrospective Case Series Study, Journal of Clinical Medicine. (2023) 12, no. 16, 10.3390/jcm12165222, 37629264. PMC10455517 37629264

[39] Margvelashvili-Malament M. , Thompson V. , Polyakov V. , and Malament K. A. , Over 14-Year Survival of Pressed e.max Lithium Disilicate Glass-Ceramic Complete and Partial Coverage Restorations in Patients With Severe Wear: A Prospective Clinical Study, Journal of Prosthetic Dentistry. (2025) 133, no. 3, 737–746, 10.1016/j.prosdent.2024.06.031, 39084921. 39084921

[40] Carvalho T. S. , Colon P. , Ganss C. , Huysmans M. C. , Lussi A. , Schlueter N. , Schmalz G. , Shellis R. P. , Tveit A. B. , and Wiegand A. , Consensus Report of the European Federation of Conservative Dentistry: Erosive Tooth Wear--Diagnosis and Management, Clinical Oral Investigations.(2015) 19, no. 7, 1557–1561, 10.1007/s00784-015-1511-7, 26121968. 26121968

[41] Tsuda Y. , Kitasako Y. , Sadr A. , Nakashima S. , and Tagami J. , Effects of Brushing Timing After Erosive Challenge on Enamel Loss In Situ: White Light Interferometer and Nanoindentation Study, Dental Materials Journal. (2016) 35, no. 4, 613–620, 10.4012/dmj.2015-405, 27477227. 27477227

[42] Hong D. W. , Lin X. J. , Wiegand A. , and Yu H. , Does Delayed Toothbrushing After the Consumption of Erosive Foodstuffs or Beverages Decrease Erosive Tooth Wear? A Systematic Review and Meta-Analysis, Clinical Oral Investigations. (2020) 24, no. 12, 4169–4183, 10.1007/s00784-020-03614-9, 33052542. 33052542

[43] Lussi A. , Buzalaf M. A. R. , Duangthip D. , Anttonen V. , Ganss C. , João-Souza S. H. , Baumann T. , and Carvalho T. S. , The Use of Fluoride For The Prevention of Dental Erosion and Erosive Tooth Wear in Children and Adolescents, European Archives of Paediatric Dentistry. (2019) 20, no. 6, 517–527, 10.1007/s40368-019-00420-0, 30762211. 30762211

[44] Petersson L. G. , The Role of Fluoride in the Preventive Management of Dentin Hypersensitivity and Root Caries, Clinical Oral Investigations. (2013) 17, no. Supplement 1, S63–S71, 10.1007/s00784-012-0916-9, 23271217. 23271217 PMC3586140

